# Metabolomic Analysis of Multiple Biological Specimens (Feces, Serum, and Urine) by ^1^H-NMR Spectroscopy from Dairy Cows with Clinical Mastitis

**DOI:** 10.3390/ani13040741

**Published:** 2023-02-19

**Authors:** Chenglin Zhu, Qian Zhang, Xin Zhao, Zhibo Yang, Falong Yang, Yang Yang, Junni Tang, Luca Laghi

**Affiliations:** 1 College of Food Science and Technology, Southwest Minzu University, Chengdu 610041, China; 2 College of Animal and Veterinary Sciences, Southwest Minzu University, Chengdu 610041, China; 3 Farming and Animal Husbandry Bureau of Ganzi County, Ganzi 626700, China; 4 Department of Agricultural and Food Sciences, University of Bologna, 47521 Cesena, Italy

**Keywords:** dairy cows, clinical mastitis, quantitative metabolome, ^1^H-NMR, pathway analysis

## Abstract

**Simple Summary:**

Dairy cows are commonly affected by mastitis, which causes huge economic losses to the dairy industry worldwide. This work aimed to study this disease by comparing multiple biological specimens (feces, serum, and urine) from individuals with or without clinical mastitis. Integrated analysis of metabolome changes across several key biofluids could facilitate knowledge discovery and reliable identification of potential biomarkers, which could in turn be used to shed light on the early diagnosis of dairy cow mastitis in its subclinical form. To the best of our knowledge, this is the first work dedicated to providing quantitative information on the multi-matrix metabolome of dairy cows with/without clinical mastitis by untargeted ^1^H-NMR. Several pathways related to energy metabolism and amino acid metabolism have been highlighted to explain the mechanisms of the changes in the dairy cow metabolome related to clinical mastitis. Our findings are the first to provide an integrated view and better understanding of the metabolic mechanism of clinical mastitis, useful for the development of regulated strategies to improve the metabolic status of mastitic dairy cows.

**Abstract:**

Due to huge economic losses to the dairy industry worldwide, mastitis can be considered as one of the most common diseases in dairy cows. This work aimed to study this disease by comparing multiple biological specimens (feces, serum, and urine) from individuals with or without clinical mastitis. This was performed by a single analytical platform, namely ^1^H-NMR, through a multi-matrix strategy. Thanks to the high reproducibility of ^1^H-NMR, we could characterize 120 molecules across dairy cow feces, serum, and urine. Among them, 23 molecules were in common across the three biofluids. By integrating the results of multi-matrix metabolomics, several pathways pertaining to energy metabolism and amino acid metabolism appeared to be affected by clinical mastitis. The present work wished to deepen the understanding of dairy cow mastitis in its clinical form. Simultaneous analysis of metabolome changes across several key biofluids could facilitate knowledge discovery and the reliable identification of potential biomarkers, which could be, in turn, used to shed light on the early diagnosis of dairy cow mastitis in its subclinical form.

## 1. Introduction

Mastitis is the most common disease affecting the mammary glands of cows, caused by an inflammation mainly triggered by pathogenic bacteria, which poses serious problems for the health of dairy cows and, in turn, for the profitability of the farms [1]. The huge economic losses caused by the disease are mainly due to the reduction in milk yield and quality, the need for expensive treatments, and even cow mortality [2,3]. Even worse, veterinary drug overuse to cure mastitis could soon pose a threat to humans, linked to antibiotic residues [4,5,6,7,8]. At the population level, the California mastitis test (CMT) is used to estimate milk somatic cell count (SCC), which is widely accepted as a convenient and rapid method in mastitis diagnosis [9]. However, several noninfectious factors, namely stage of lactation, season, milking frequency, and parity, can affect SCC, leading to false-positive results [10,11]. Furthermore, it is still controversial to establish the exact cutoff value to differentiate the cows with healthy udders from mastitic cows [12]. Therefore, additional diagnosis methods or potential biomarker discoveries would be helpful to enhance the accuracy of the detection of cow mastitis.

Metabolomics, being downstream of genomics, transcriptomics, and proteomics, can provide insight into the phenotype of a biofluid, which provides a platform for the discovery of biomarkers of a disease and for the identification of the metabolic pathways it triggers. Taking advantages of univariate/multivariate analysis, it can work on the small weight metabolites concentrations directly, and then integrate consequences of external stimuli [13]. Among the techniques for metabolomic investigations, ^1^H-NMR spectroscopy is considered one of the most used, because it is based on very simple procedures for sample preparation, it is intrinsically non-invasive and strongly quantitative, and it is characterized by high reproducibility and superb instrument stability, which counterbalance a sensitivity lower than other platforms, such as GC-MS [14]. For these reasons, ^1^H-NMR spectroscopy has been used in studies about domestic animals to acquire metabolomic profiles of numerous biofluids, for instance urine [15], plasma [16], serum [17], feces [18], tracheal wash, exhaled breath condensate [19], and seminal plasma [20].

Up to now, metabolomics based on ^1^H-NMR have been widely applied for in-depth characterization of the metabolic differences between cows with mastitis and their healthy counterparts, particularly powerful in identifying metabolites associated with increased risk of clinical mastitis. For instance, by means of ^1^H-NMR, Bobbo et al. found that milk samples with high somatic cells showed lower concentrations of dimethyl sulfone, riboflavin, galactose-1-phosphate, galactose, carnitine, glucose, hippurate, lactose, orotate, succinate, and lecithin, together with higher contents of O-acetylcarnitine, lactate, choline, 2-oxoglutarate, phenylalanine, valine, and acetate [21]. 

Previous studies looking for metabolic fingerprints of mastitis have already provided promising results linked to the detection of biomarkers and pathway clarification. Unfortunately, the clear majority of these studies tended to focus on a single biological matrix, so that they may have missed relevant molecules pertaining to complementary physiological pathways, covered by other matrices. In detail, the serum metabolome can provide information over a large range of biochemical processes, because it contains both polar and non-polar metabolites [22]. Urine usually includes metabolic breakdown products, derived from a wide range of foods, endogenous waste metabolites, environmental contaminants, and bacterial by-products [23]. The metabolome of feces is the most efficient in capturing the complex interactions between the gut microbiome and the host. Indeed, there is an increasing awareness that the gut microbiota plays a crucial role in maintaining the physiological homeostasis of a host [24]. In particular, the entero-mammary pathway theory establishes a possible connection between endogenous gut microbiota and the development of clinical mastitis [25].

Up to now, the analysis of molecules in different biofluids of a single animal has only been applied in a restricted number of studies. In addition, limited information is available about the typical concentration ranges of major molecules in the biofluids of dairy cows with or without mastitis. To address these issues, we propose an investigation strategy based on multi-matrix metabolomics, by analyzing multiple biological specimens (feces, serum, and urine) from the same individual on a single analytical platform. The present work could deepen our understanding of dairy cow mastitis. Moreover, simultaneous analysis of metabolomic changes through several biofluids could facilitate knowledge discovery and reliable potential biomarker identification and could shed light on the early diagnosis of dairy cow subclinical mastitis.

## 2. Materials and Methods

### 2.1. Sampling

The experimental designs and protocols of the current study were approved by Southwest Minzu University Animal Ethics Committee (protocol NO. SWUN-A-0045) and were in accordance with the recommendations of the academy’s guidelines for animal research. Thirteen Chinese Holstein cows were enrolled in the work, six healthy and seven with clinical mastitis. All the samples were obtained at a pasture in Chengdu, Sichuan, in routine physical examinations. In particular, venous blood samples were obtained through syringe with the help of an experienced veterinarian.

In free housing systems, following the standard practices, all cows involved in the study were fed with total mixed rations (TMR) during their indoor period. The dairy cows were milked twice a day. All the samples analyzed were refrigerated right after collection and transported under ice to the lab in less than two hours.

### 2.2. Metabolomic Analysis

Feces, serum, and urine sample preparations for ^1^H-NMR analysis were conducted in accordance with Zhu et al. [18]. In brief, we took 80 mg stool with 1 mL of deionized water in an Eppendorf tube and vortex mixed it for 5 min, followed by centrifuging for 15 min at 18,630 g and 4 °C. Then, we moved 0.7 mL of supernatant to a new Eppendorf tube with 0.2 mL of NMR analysis solution added before. An amount of 1 mL of serum samples were thawed and centrifuged at the above conditions. Then, the supernatant (0.5 mL) was mixed with NMR analysis solution (0.1 mL). Similar to serum samples, we thawed and centrifuged urine samples at the above conditions. An amount of 0.35 mL of supernatant was mixed with 0.35 mL of bi-distilled water and 0.2 mL of NMR analysis solution. Finally, each of the obtained samples was centrifuged again at the above conditions just before analysis.

^1^H-NMR spectra were recorded at 298 K with an AVANCE III spectrometer (Bruker, Wuhan, China) operating at a frequency of 600.13 MHz. Following Zhu et al. [26], the signals from broad resonances originating from large molecules were suppressed by a CPMG-filter composed of 400 echoes with a τ of 400 μs and a 180° pulse of 24 μs, for a total filter of 330 ms. The HOD residual signal was suppressed by means of presaturation. This was conducted by employing the cpmgpr1d sequence, part of the standard pulse sequence library. Each spectrum was acquired by summing up 256 transients using 32 K data points over a 7184 Hz spectral window, with an acquisition time of 2.28s. In order to apply NMR as a quantitative technique [27], the recycle delay was set to 5s, keeping into consideration the relaxation time of the protons under investigation. ^1^H-NMR spectra were baseline-adjusted by means of peak detection according to the “rolling ball” principle [28] implemented in the baseline R package [29]. In order to get rid of changes in water and fiber contents among samples, we applied probabilistic quotient normalization (PQN) [30] to the entire spectra array. Molecule identification was set up through comparing their multiplicity and chemical shift according to the library of Chenomx (Chenomx Inc., Edmonton, AB, Canada, ver 8.4).

### 2.3. Statistical Analysis

We used R computational language to perform statistical analysis [31]. Student t-test was used to find molecules whose concentration altered between groups. To fulfill the requirements, a *p*-value below 0.05 was accepted. Prior to the t-test, not-normally distributed data were transformed taking advantage of Box and Cox by means of “MASS” package in R [32].

In order to address the overall trends of the metabolomic features of the samples, a principal component analysis model in the robust version (rPCA) was set up through the “agricolae” package [33], on the basis of the molecules selected by t-test. For the model, the scoreplot was calculated to highlight the structure of the data. Moreover, the Pearson correlation plot was performed to underline the relations between molecule concentrations and model components.

We performed pathway analysis by means of MetaboAnalyst 5.0 [34]. Combining the pathway enrichment analysis results, we could identify the most relevant pathways [35]. For the purpose, only molecules whose concentration resulted as significantly different in the t-test were involved. 

## 3. Results

### 3.1. Characterization of Molecules in Feces, Serum, and Urine

In the present work, we were able to characterize 120 molecules across dairy cow feces, serum, and urine, giving information about diet, protein digestion, energy generation, and gut-microbial co-metabolism, sorted to several chemical groups, namely amino acids, peptides and analogues, nucleosides, nucleotides and analogues, carbohydrates and derivates, organic acids and derivates, and miscellaneous.

The entire list of molecules and their quantifications are shown in Tables S2–S4. Typical ^1^H-NMR spectra from feces, serum, and urine are reported in Figures S1–S3, respectively. In detail, we identified and quantified 67 molecules in feces, 54 in serum, and 77 in urine. Among the molecules characterized, 23 metabolites were identified across the three biofluids. Moreover, 14, 14, and 35 metabolites were unique to feces, serum, and urine, respectively (Figure 1). Signals from ^1^H-NMR were assigned as pictorially described in Figures S4–S33.

### 3.2. Feces Metabolome Affected by Clinical Mastitis

Sixteen molecules were significantly distinct between the two groups, namely glycine, O-acetylcholine, glutamine, O-phosphocholine, benzoate, tyrosine, creatine, methanol, valerate, 1,3-dihydroxyacetone, propionate, acetate, pyruvate, acetoacetate, 2,3-butanediol, and ethanol, as shown in Table 1.

**Figure 2:**
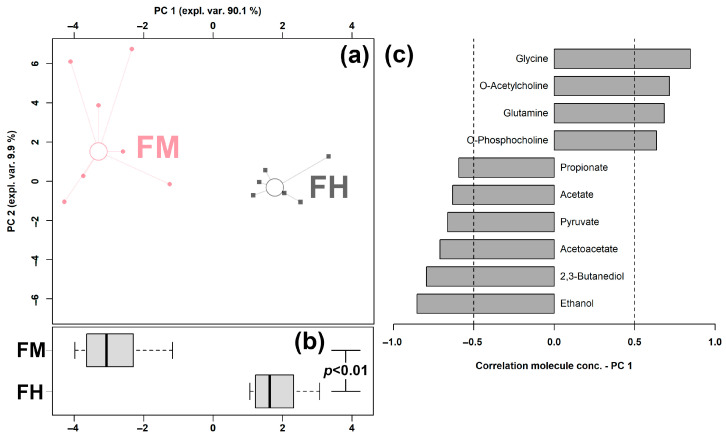
rPCA model was set up on the basis of the molecules whose concentrations showed significantly distinct between groups. In the scoreplot (**a**), we use squares (Healthy) and circles (Clinical Mastitis) to show the samples from the two groups, respectively. The median of each samples’ group is indicated by wide, empty circles. Boxplot (**b**) highlights the position of the samples along PC 1. The loading plot (**c**) shows the significant relationships between the concentration of each molecule and its importance over PC 1 (*p* < 0.05).

In order to obtain an overview of the trends of the so-evidenced molecules, their concentrations were employed as a basis for an rPCA model, shown in Figure 2.

In the scoreplot, as shown in Figure 2a, the PC 1 accounted for as high as 90.1% of the overall samples’ variability, perfectly underlining the distinct features between the two groups. In particular, feces from clinical mastitic cows were found to be mainly characterized by higher levels of propionate, acetate, pyruvate, acetoacetate, 2,3-butanediol, and ethanol and lower amounts of glycine, O-acetylcholine, glutamine, O-phosphocholine, benzoate, tyrosine, and creatine.

### 3.3. Serum Metabolomic Features Affected by Clinical Mastitis

Seven of the molecules quantified in serum were significantly altered between groups, namely 3-methylhistidine, asparagine, citrate, formate, lactate, phenylalanine, and serine, as shown in Table 2.

**Figure 3:**
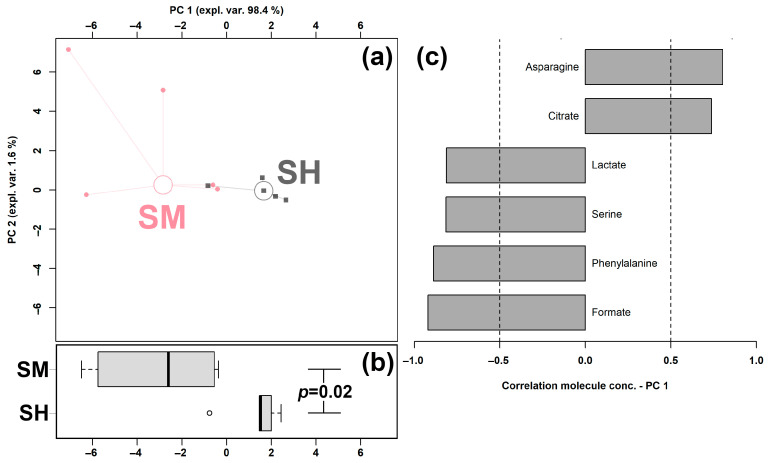
rPCA model was set up on the basis of the molecules whose concentrations showed significantly distinct between groups. In the scoreplot (**a**), we use squares (Healthy) and circles (Clinical Mastitis) to show the samples from the two groups, respectively. The median of each samples’ group is indicated by wide, empty circles. Boxplot (**b**) highlights the position of the samples along PC 1. The loading plot (**c**) shows the significant relationships between the concentration of each molecule and its importance over PC 1 (*p* < 0.05).

In order to obtain an overview of the trends of the so-evidenced molecules, their concentrations were employed as a basis for an rPCA model, shown in Figure 3.

In the scoreplot, as shown in Figure 3a, the PC 1 accounted for as high as 98.4% of the overall samples’ variability, perfectly underlining the differences between the two groups. For instance, serum samples obtained from cows with clinical mastitis were investigated to be mainly characterized by higher amounts of lactate, serine, phenylalanine, and formate and lower concentrations of asparagine and citrate.

### 3.4. Urine Metabolome Affected by Clinical Mastitis

**Figure 4:**
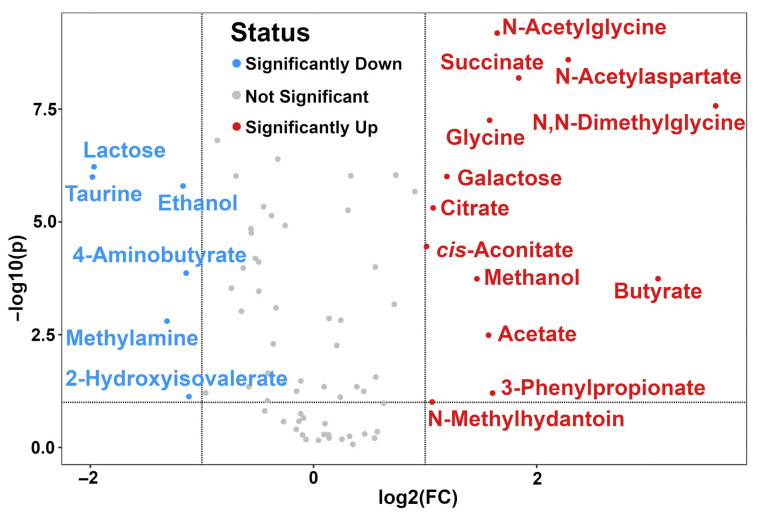
Volcano plot indicating the changes in the concentrations of metabolites in urine samples from healthy and clinical mastitic dairy cows.

Fifty-five metabolites were altered between the two groups significantly, namely creatine, O-acetylcarnitine, fumarate, citrate, trimethylamine N-oxide, lactose, N-acetylglucosamine, 2-oxoglutarate, glycine, choline, cis-aconitate, creatinine, proline, 2-oxoglutarate, citrate, leucine, tyrosine, lactate, valine, carnitine, dimethylamine, arginine, maltose, and isoleucine. According to Zhu et al., a volcano plot was set up, which nicely presented the results of both t-test and fold change analysis [36]. Significantly different molecules with a fold change higher than 2 are shown in Figure 4. 

Compared to dairy cows with clinical mastitis, healthy individuals exhibited higher concentrations of N-acetylglycine, succinate, N-acetylaspartate, N,N-dimethylglycine, glycine, galactose, citrate, cis-aconitate, methanol, butyrate, acetate,3-phenylpropionate, and N-methylhydantoin, and lower levels of lactose, taurine, ethanol, 4-amonobutyrate, methylamine, and 2-hydroxyisovalerate.

### 3.5. Pathway Analysis in Relation to Clinical Mastitis

In order to identify the most relevant pathways differentiating the groups, a pathway enrichment analysis was performed on the basis of molecules whose concentration was significantly altered between the two groups. 

**Figure 5:**
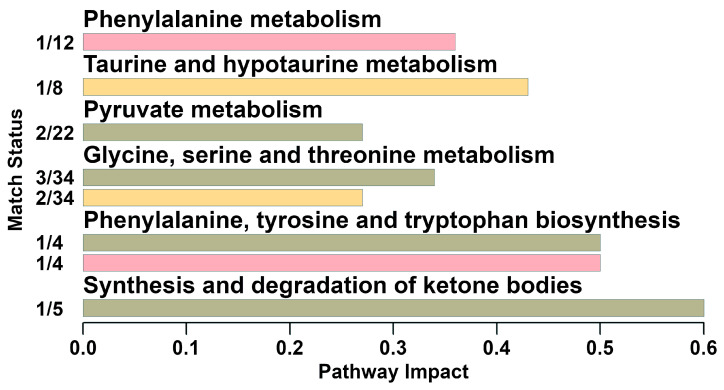
Enrichment analysis based on the biofluids’ metabolites whose concentrations showed significantly different between groups (the cut-off of impact value was above 0.2).

Six pathways were addressed, namely phenylalanine metabolism, glycine, serine and threonine metabolism, taurine and hypotaurine metabolism, pyruvate metabolism, phenylalanine, tyrosine and tryptophan biosynthesis, and synthesis and degradation of ketone bodies (Figure 5).

## 4. Discussion

In recent decades, how to diagnose and cure dairy cow mastitis in its early stages has become one of the key points for the entire dairy chain. Such issue is of great interest not only to farmers and processing industries, but also to consumers, as this would increase sensitivity and awareness about animal health and welfare [37]. Up to now, researchers have paid most of their attention to the changes in the milk metabolome as a consequence of mastitis [9,38], while only a few works focused on other biofluids, such as feces [39], serum [40,41], and urine [42]. Furthermore, rare information refers to the evaluation of disturbances of multi-biofluid metabolomic profiles affected by mastitis, even though it has been confirmed that this choice can provide a comprehensive view of the response of the animal to internal and external stimuli [18,43,44]. In order to fill these gaps, this is the first attempt to profile the metabolites of three different biofluids (feces, serum, and urine) simultaneously from the same dairy cows, with or without mastitis, by means of a single platform, ^1^H-NMR. Taking advantage of its quantitative character, 23 molecules were observed across the three biofluids, while 14, 14, and 35 molecules were observed exclusively in feces, serum, and urine, respectively. The unique compounds identified in each of the three biofluids are in line with previous results and are typical of the three different biofluids in dairy cows [43]. In addition, we found that urine had the most varied metabolomic profile, compared to the other two biofluids, which is in agreement with Sun et al. [45]. 

In feces, concentrations of sixteen molecules resulted as significantly different between mastitic and healthy individuals. Among them, ten molecules were highlighted by an rPCA model, as their concentrations were significantly correlated with PC 1. Propionate, acetate, pyruvate, acetoacetate, 2,3-butanediol, and ethanol appeared as more concentrated in mastitic cows, while glycine, O-acetylcholine, glutamine, and O-phosphocholine appeared as less concentrated. Interestingly, all the molecules exhibiting higher levels in mastitic dairy cows were related to energy metabolism. Acetoacetate could serve as an indispensable extrahepatic source of energy. Moreover, it has the possibility to provide acetoacetyl-CoA and acetyl-CoA for the synthesis of cholesterol, fatty acids, and complex lipids. 2,3-Butanediol could be generated by the anaerobic fermentation of glucose by gut microbiota. Ethanol is metabolized by the body as an energy-providing nutrient, as it evolves into acetyl-CoA, an intermediate in common with glucose metabolism that can be used for energy in the citric acid cycle or for biosynthesis. Almost all of the dietary carbohydrates are fermented to volatile fatty acids (acetate, propionate, and butyrate) in the rumen of the dairy cows, with propionate as the predominant substrate for gluconeogenesis [46]. Pyruvate, which is the starting point of gluconeogenesis and the end product for glycolysis [47], can be considered as an important intermediate metabolite for the generation of propionate from the succinic pathway or the lactate pathway [48]. It can be converted by the pyruvate dehydrogenase complex into acetyl-CoA, which can then enter the TCA cycle. The TCA cycle plays a central role in cellular respiration and the supply of energy to all living cells [49], which is of paramount significance to the cell’s metabolic efficiency and, therefore, to the cow’s metabolism and production [50]. In the present study, such results suggest that the energy-related metabolic pathways were altered due to mastitis. Subsequently, it can lead to decreased milk production [51]. 

Glycine is biosynthesized in the body from several amino acids, such as serine and threonine. Moreover, it can be derived from 3-phosphoglycerate, choline, or hydroxyproline via inter-organ metabolism of the liver and kidneys. Glutamine is derived from the pathway of glutamate metabolism. The metabolic pathways of aspartate and glutamate produce glutamine and succinate [52]. In the present work, a lower level of glutamine was found in dairy cows with mastitis, compared to healthy individuals, in line with Wang et al. [39]. There is a significant body of evidence that links glutamine-enriched diets with positive intestinal effects [53]. These include maintenance of gut barrier function and aiding intestinal cell proliferation and differentiation. Both O-acetylcholine and O-phosphocholine have been found to be microbial products of choline, which is an essential metabolite and can be involved in many biological processes, in particular, lipid metabolism and cell signaling. Therefore, we speculate that mastitis could have an adverse effect on gut microbiota and intestinal function, mainly through glycine, serine, and threonine metabolism pathways, as indicated by pathway analysis. 

Serum has been considered as the optimal biofluid for potential biomarker discovery to predict mastitis, due to the multiple metabolites that were proven to be altered 8 weeks before subclinical mastitis [54]. In the current study, the serum metabolome was altered by clinical mastitis mainly in its amino acids’ and organic acids’ fractions. In detail, dairy cows with clinical mastitis exhibited higher levels of lactate, serine, phenylalanine, and formate, while lower amounts of asparagine and citrate. The above results were in line with previous studies [41,55]. Asparagine could be converted from glutamine through the asparagine synthetase enzyme. Citrate is formed in the TCA cycle or obtained through diet and participates as an intermediate in the metabolic oxidation of carbohydrates in animal tissues. Lower levels of citrate may suggest a retardation of energy metabolism connected to mastitis [56]. Lactate is considered as the main end product of carbohydrates’ metabolism [57]. Serine plays an important role in the process of methylation, originating from key metabolic intermediates such as creatine, phosphatidylcholine, and epinephrine to methylation of proteins, DNA, and RNA [58]. In addition, one of serine’s functions is to help the synthesis of phospholipids, which are part of cell membranes [59]. Konashi et al. demonstrated that an inadequate intake of dietary serine lowers the immune response in chickens [60]. Therefore, we could speculate that the elevated concentration of serine is an indicator of the activation of immune response in cows with clinical mastitis. Phenylalanine is an essential amino acid and the precursor of catecholamines, which are neurotransmitters and adrenaline-like substances [61]. This molecule is transformed into tyrosine through the action of phenylalanine hydroxylase and a biopterin cofactor [62]. In serum of dairy cows with mastitis, the elevated levels of phenylalanine and serine suggested that mastitis was preceded and followed by alteration in amino acid metabolism, pertaining to phenylalanine metabolism and phenylalanine, tyrosine, and tryptophan biosynthesis. Overall, the results of pathway analysis, in line with Sun et al. [63], suggest that the pathways associated with amino acid metabolism may grant a reservoir of biomarkers for higher milk yield and better milk protein quality. 

In urine, citrate has been confirmed as a potential biomarker of the metabolic difference between lactation and non-lactation [45]. The excretion of lactose in the urine has appeared as a reliable marker of changes occurring in breast permeability, caused by the inflammation of the breast [64]. In our study, the significantly higher amount of lactose excreted is most likely explained by an increase in permeability of the paracellular pathway, related to an oversupply of milk [64,65]. Similar to serine in serum, taurine is reported to have a key role in the regulation of the immune response, thus reducing tissue damage induced by bacterial infection. Miao et al. found that taurine can be used to regulate the immune response following infection by S. uberis and consequently prevent mammary tissue damage by increasing Treg cells in a rat model [66]. Methylamine is an amino acid produced in the process of rumen nitrogen metabolism [43]. N-acetylaspartate is synthesized from aspartate and acetyl-CoA. The importance of N-acetylaspartate is related to the fact that it supplies acetate groups for the synthesis of acetyl-CoA and acetylation of proteins that are important for immune responses [67]. Acetyl groups deriving from N-acetylaspartate are used for acetylation of transcription factor NF-kB, which mediates the production of pro-inflammatory cytokines such as the production of TNF by macrophages. Dervishi et al. found that excretion of N-acetylaspartate in the urine might be helpful to ease the process of inflammation [68]. Galactose, as a carbohydrate, is considered as an important source of energy for dairy cows. It is the precursor of fat and lactose in cows’ milk. Greater elimination of carbohydrates in the urine might suggest that clinical mastitic cows have an impaired energy utilization. 3-Phenylpropionate is a vital ruminal aromatic acid. Studies supplying varying levels of grain to lactating dairy cows reported positive correlations between acetate and 3-phenylpropionate and rumen pH. Interestingly, acetate and 3-phenylpropionate were correlated with liveweight [69]. Lower levels of 3-phenylpropionate in dairy cows with mastitis may suggest that not only gut microbiota, but also the rumen environment, in particular pH, could be altered by clinical mastitis.

## 5. Conclusions

To the best of our knowledge, this is the first work devoted to providing the quantitative information of the dairy cow multi-matrix metabolome with/without clinical mastitis by means of untargeted ^1^H-NMR. Several pathways pertaining to energy metabolism and amino acid metabolism were addressed to explain the mechanisms of dairy cow metabolome variation affected by clinical mastitis. Our findings are the first to provide an integrated insight and a better understanding of the metabolic mechanism of clinical mastitis, which is beneficial for developing regulated strategies to improve the metabolic status of mastitic dairy cows.

## Figures and Tables

**Figure 1:**
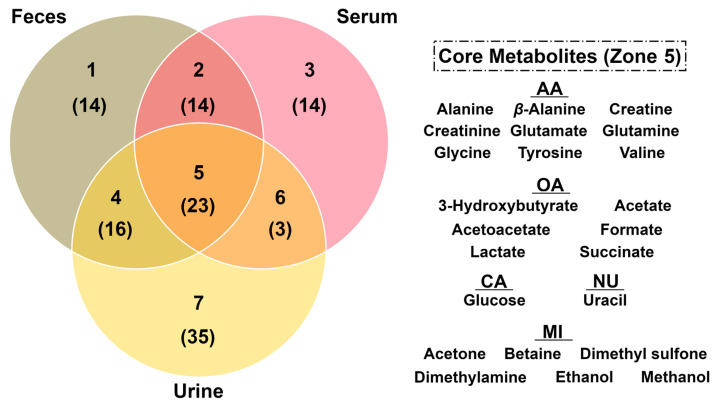
Venn diagram showing unique and shared metabolites among dairy cow feces, serum, and urine. Each number represents a zone of intersection, while the numbers in brackets indicate the number of metabolites comprised in the specified zone (see Table S1 for the corresponding list of molecules). The list of core metabolites (zone 5) is shown on the right, divided in amino acids, peptides and analogues (AA), organic acids and derivates (OA), carbohydrates and derivates (CA), nucleosides, nucleotides and analogues (NU), and miscellaneous metabolites (MI).

**Table 1:** Concentrations (mmol/g, mean ± sd) in feces of molecules significantly different between healthy and clinical mastitic cows.

	Healthy (FH)	Clinical Mastitis (FM)	*p*-Value	Trend
1,3-Dihydroxyacetone	4.87 × 10^−5^ ± 1.25 × 10^−5^	6.63 × 10^−5^ ± 1.48 × 10^−5^	^#^ 0.040 *	↑
2,3-Butanediol	4.81 × 10^−5^ ± 1.87 × 10^−5^	1.39 × 10^−4^ ± 6.12 × 10^−5^	0.001 **	↑
Acetate	4.61 × 10^−2^ ± 4.71 × 10^−3^	6.14 × 10^−2^ ± 1.55 × 10^−2^	0.046 *	↑
Acetoacetate	2.44 × 10^−5^ ± 5.81 × 10^−6^	3.47 × 10^−5^ ± 7.09 × 10^−6^	0.015 *	↑
Benzoate	5.91 × 10^−4^ ± 4.19 × 10^−4^	2.16 × 10^−4^ ± 1.97 × 10^−4^	0.011 *	↓
Creatine	4.68 × 10^−5^ ± 9.63 × 10^−6^	4.20 × 10^−5^ ± 7.00 × 10^−5^	0.031 *	↓
Ethanol	1.01 × 10^−4^ ± 2.20 × 10^−5^	1.60 × 10^−4^ ± 3.84 × 10^−5^	0.006 **	↑
Glutamine	1.95 × 10^−4^ ± 6.22 × 10^−5^	1.28 × 10^−4^ ± 2.61 × 10^−5^	0.047 *	↓
Glycine	4.14 × 10^−4^ ± 8.73 × 10^−5^	1.70 × 10^−4^ ± 2.69 × 10^−5^	0.000 ***	↓
Methanol	1.20 × 10^−4^ ± 1.75 × 10^−5^	2.11 × 10^−4^ ± 1.58 × 10^−4^	0.028 *	↑
O-Acetylcholine	1.02 × 10^−5^ ± 1.87 × 10^−6^	6.45 × 10^−6^ ± 2.33 × 10^−6^	0.008 **	↓
O-Phosphocholine	9.84 × 10^−6^ ± 2.16 × 10^−6^	6.75 × 10^−6^ ± 2.51 × 10^−6^	0.036 *	↓
Propionate	8.74 × 10^−3^ ± 9.20 × 10^−4^	1.27 × 10^−2^ ± 3.77 × 10^−3^	0.020 *	↑
Pyruvate	8.19 × 10^−6^ ± 2.51 × 10^−6^	1.26 × 10^−5^ ± 3.15 × 10^−6^	0.016 *	↑
Tyrosine	9.88 × 10^−5^ ± 1.52 × 10^−5^	7.95 × 10^−5^ ± 1.62 × 10^−5^	0.049 *	↓
Valerate	8.92 × 10^−4^ ± 1.15 × 10^−4^	1.41 × 10^−3^ ± 4.98 × 10^−4^	0.009 **	↑

^#^ “*”, “**”, and “***” represent *p*-values below 0.05, 0.01, and 0.001, respectively.

**Table 2:** Concentrations (mmol/g, mean ± sd) in serum of the molecules significantly different between healthy and clinical mastitic cows.

	Healthy (SH)	Clinical Mastitis (SM)	*p*-Value	Trend
3-Methylhistidine	2.81 × 10^−1^ ± 3.28 × 10^−2^	3.49 × 10^−1^ ± 3.59 × 10^−2^	^#^ 0.014 *	↑
Asparagine	1.64 × 10^−1^ ± 1.98 × 10^−2^	1.35 × 10^−1^ ± 1.49 × 10^−2^	0.036 *	↓
Citrate	5.29 × 10^−1^ ± 5.32 × 10^−2^	2.80 × 10^−1^ ± 1.35 × 10^−1^	0.011 *	↓
Formate	1.06 × 10^−1^ ± 2.89 × 10^−2^	1.54 × 10^−1^ ± 4.68 × 10^−2^	0.048 *	↑
Lactate	5.31 ± 9.79 × 10^−1^	12.10 ± 7.25	0.029 *	↑
Phenylalanine	6.09 × 10^−1^ ± 1.02 × 10^−1^	8.70 × 10^−1^ ± 2.32 × 10^−1^	0.035 *	↑
Serine	3.99 × 10^−1^ ± 2.63 × 10^−2^	4.97 × 10^−1^ ± 1.21 × 10^−1^	0.045 *	↑

^#^ “*” represents *p*-value below 0.05.

## Data Availability

None of the data were deposited in an official repository. The data that support the study findings are available upon request.

## Supplementary Materials

The following supporting information can be downloaded at: https://www.mdpi.com/article/10.3390/ani13040741/s1.
