# Supplementary material for: Metabolomic Analysis of Multiple Biological Specimens (Feces, Serum, and Urine) by 1H-NMR Spectroscopy from Dairy Cows with Clinical Mastitis
Source: Animals (Basel). 2023 Feb 19;13(4):741. doi: 10.3390/ani13040741 (PMC9952568; doi:10.3390/ani13040741)
Supplement: Supplementary file 1 [file animals-13-00741-s001.zip › supplementary figures.pdf]

Metabolomic Analysis of Multiple Biological Specimens (Feces, Serum, and Urine) by ^{1}H-NMR Spectroscopy from Dairy Cows with Clinical Mastitis

Chenglin Zhu [1], Qian Zhang [1], Xin Zhao [1], Zhibo Yang [1], Falong Yang [2], Yang Yang [3], Junni Tang [1,*] and Luca Laghi [4,*]

Supplementary material

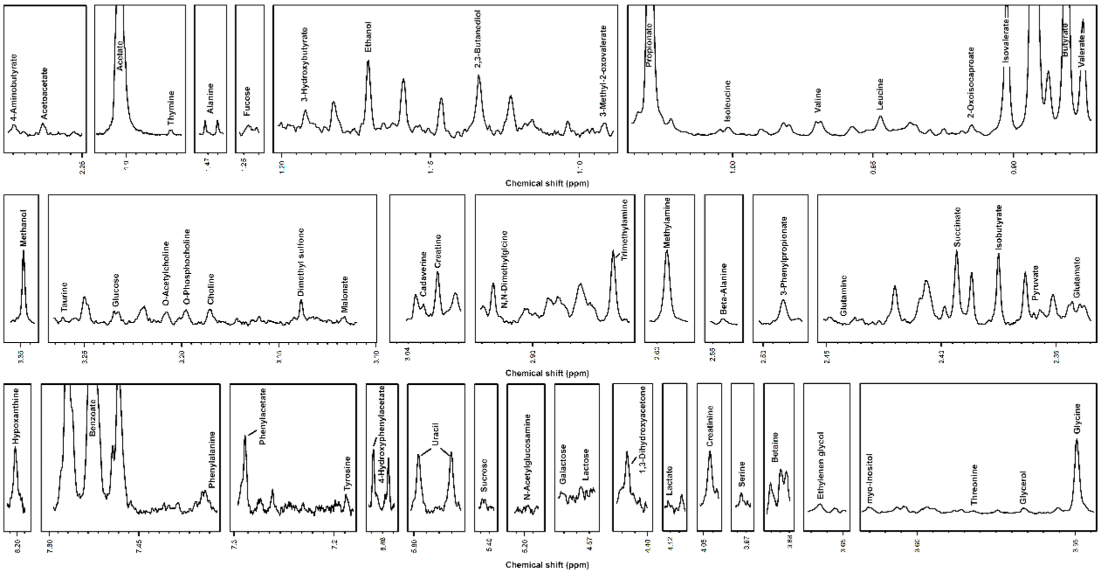

Figure S1. ^{1}H-NMR spectrum from feces sample representative of all the registered spectra. The name of each molecule appears over the signal used for its quantification. To ease the reader's visual inspection, for each portion a spectrum with a convenient signal-to-noise ratio has been selected.

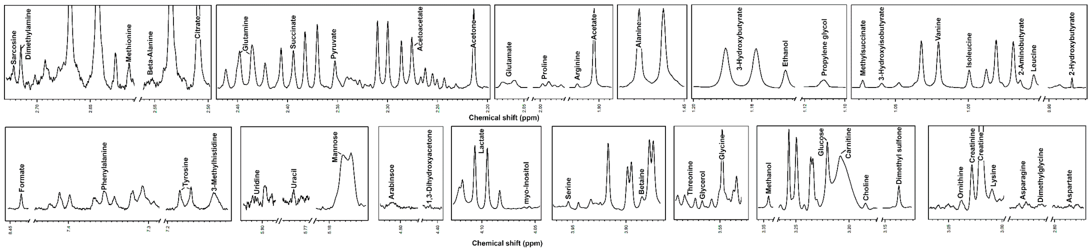

Figure S2. ^{1}H-NMR spectrum from serum sample representative of all the registered spectra. The name of each molecule appears over the signal used for its quantification. To ease the reader's visual inspection, for each portion a spectrum with a convenient signal-to-noise ratio has been selected.

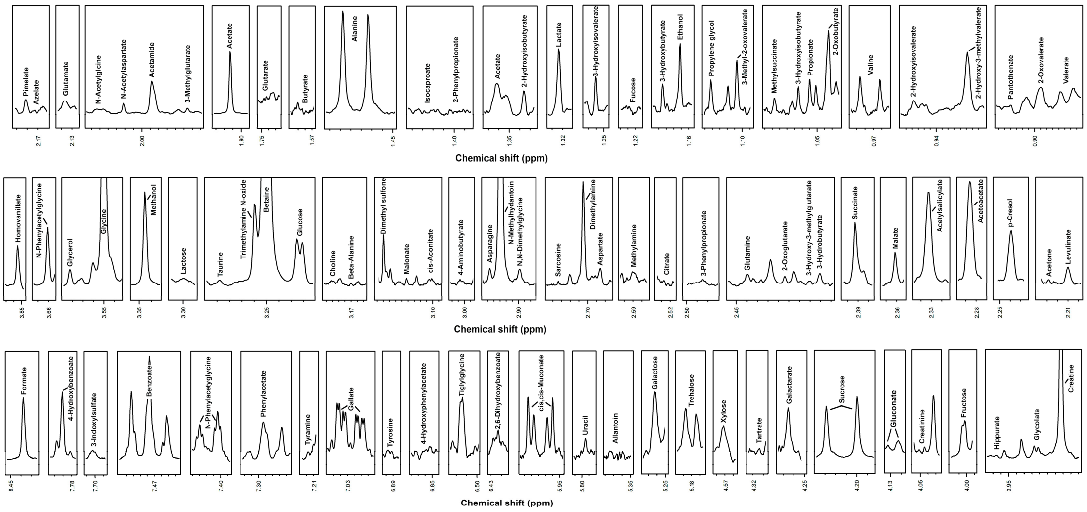

Figure S3. ^{1}H-NMR spectrum from urine sample representative of all the registered spectra. The name of each molecule appears over the signal used for its quantification. To ease the reader's visual inspection, for each portion a spectrum with a convenient signal-to-noise ratio has been selected.

Molecules assignment and quantification

Figures S4-S33: Pictorial description of the molecules' assignment and quantification procedure by Chenomx software. Upper panel - portions of the spectra in white-washed mode. Lower panel – One representative registered spectrum (black line) superimposed to the signals simulated by Chenomx software (red line) for each of the molecules listed. The black and red dashed lines evidence the signals used for quantification purposes.

Feces

Figure S4

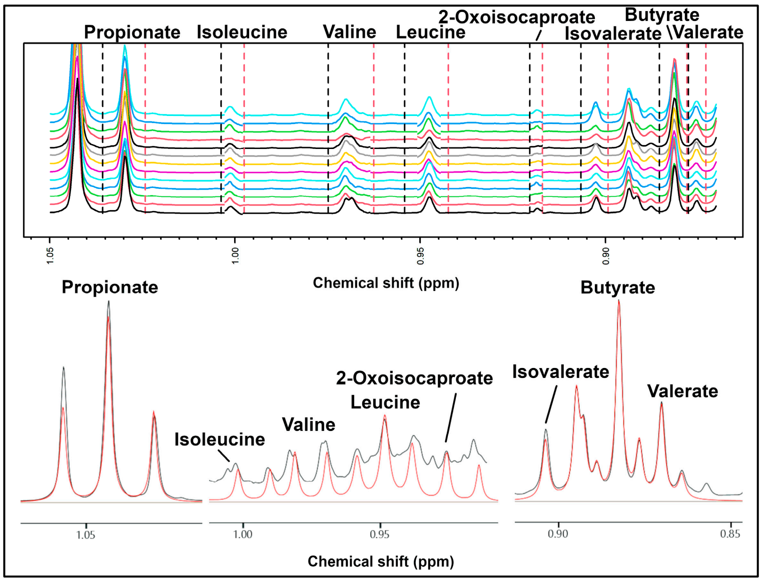

Figure S5

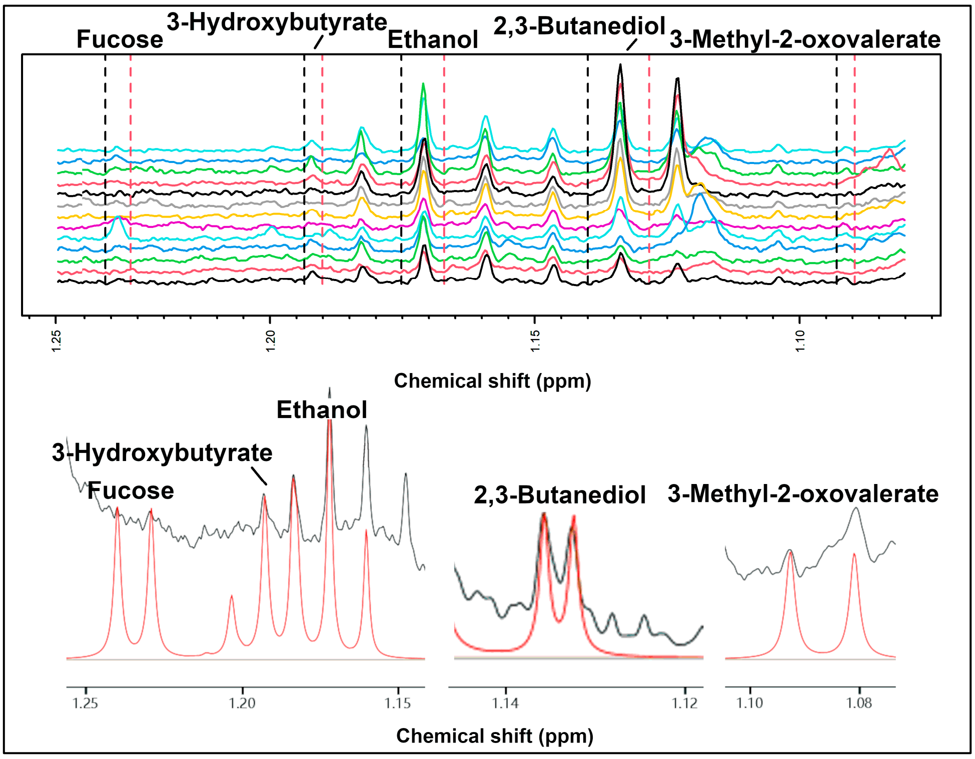

Figure S6

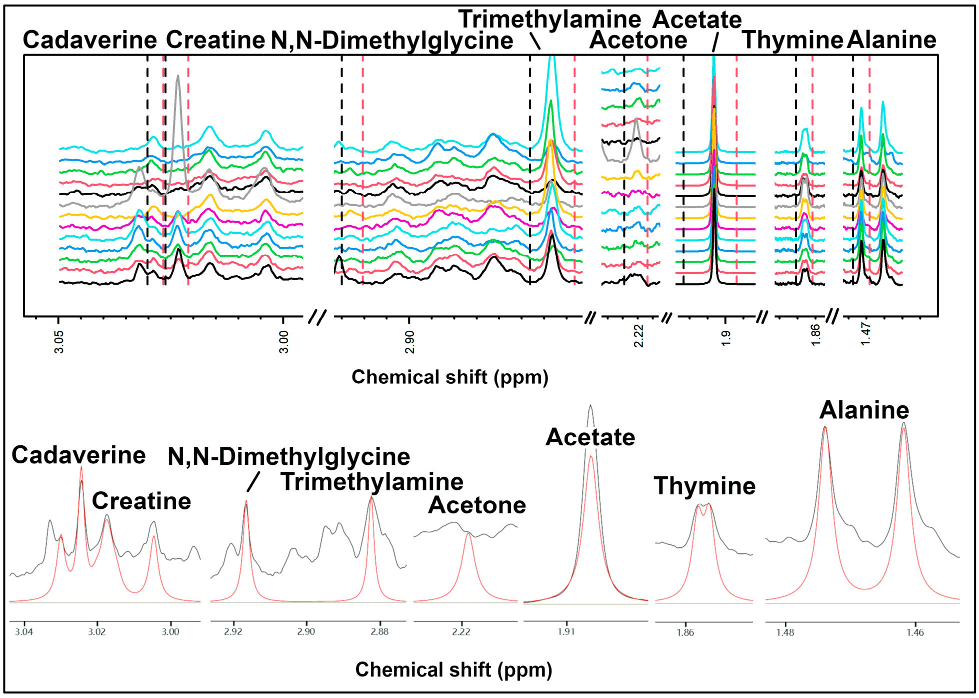

Figure S7

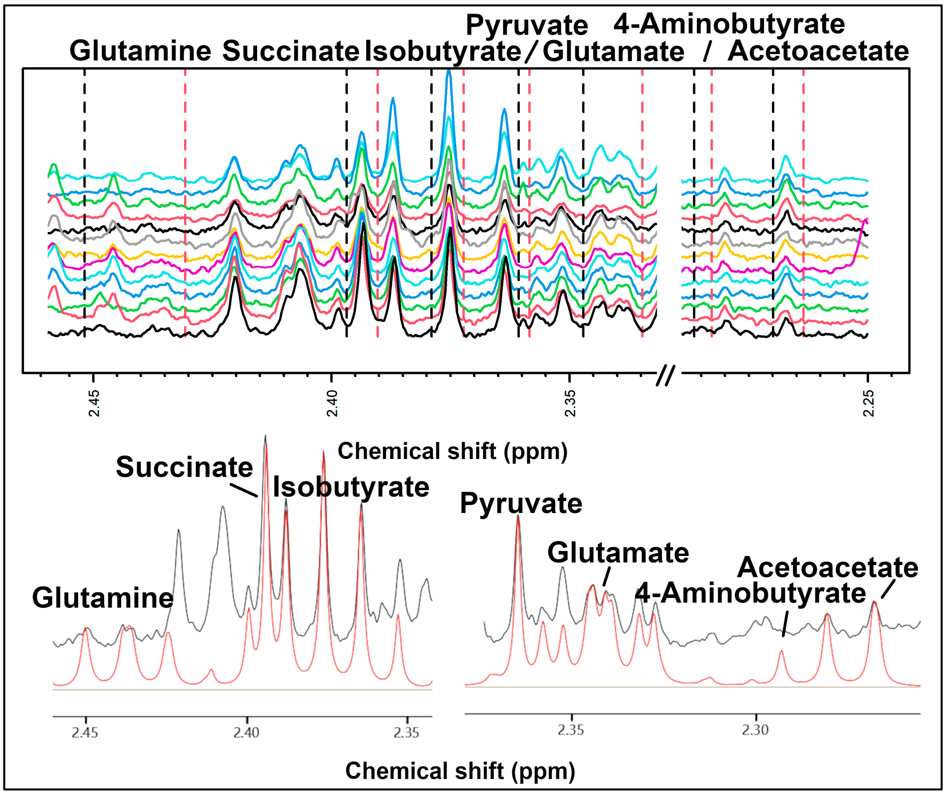

Figure S8

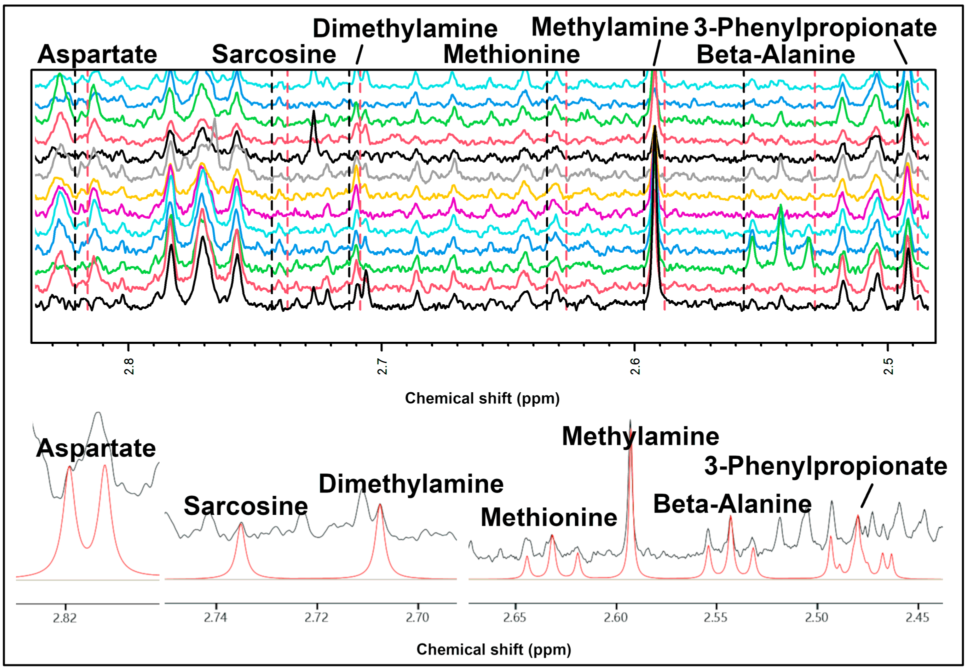

Figure S9

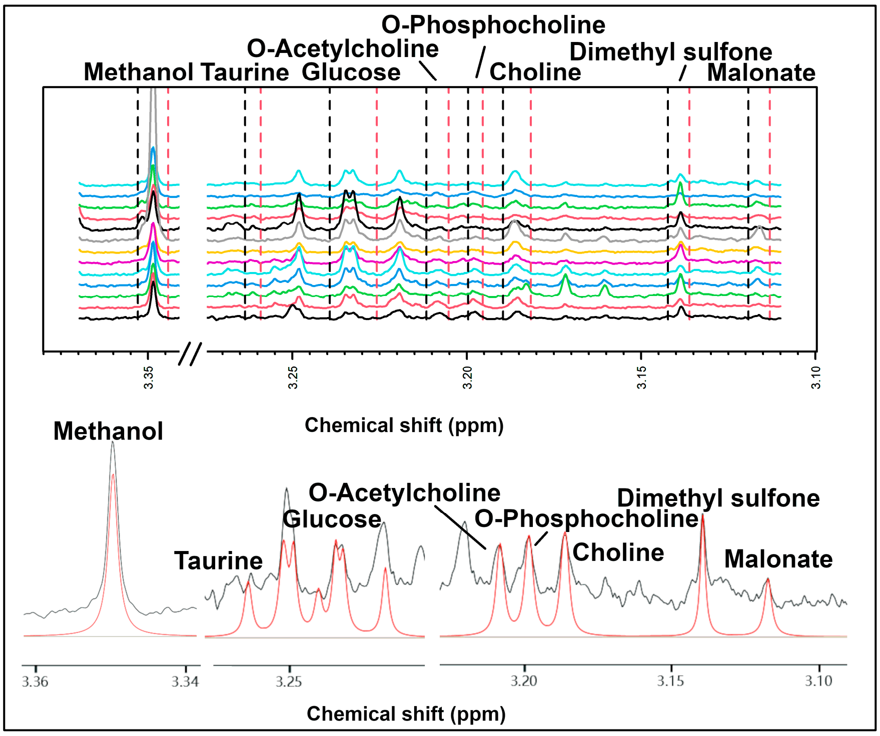

Figure S10

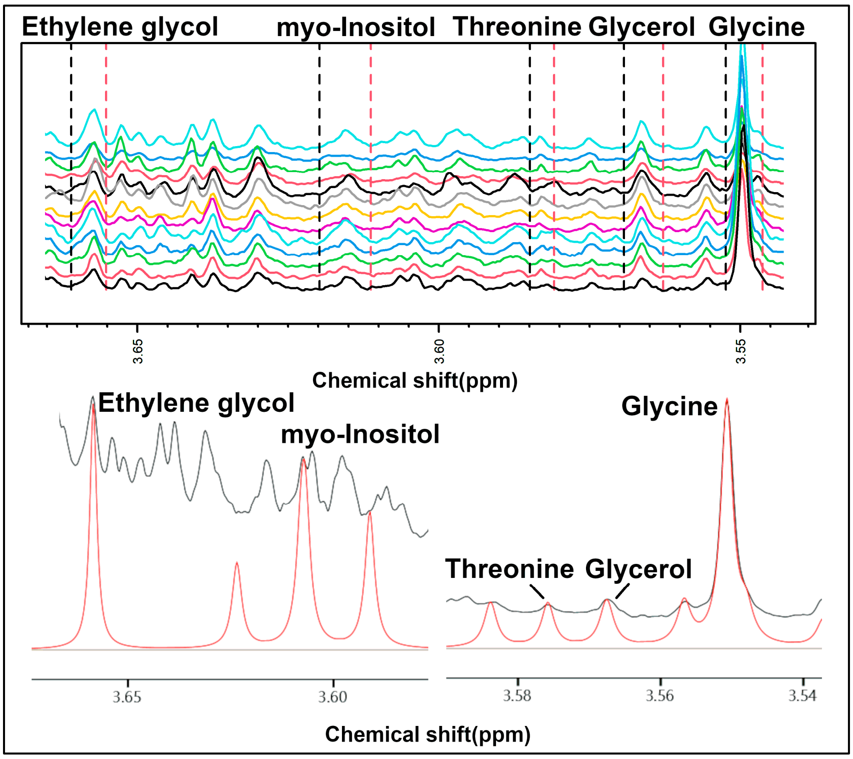

Figure S11

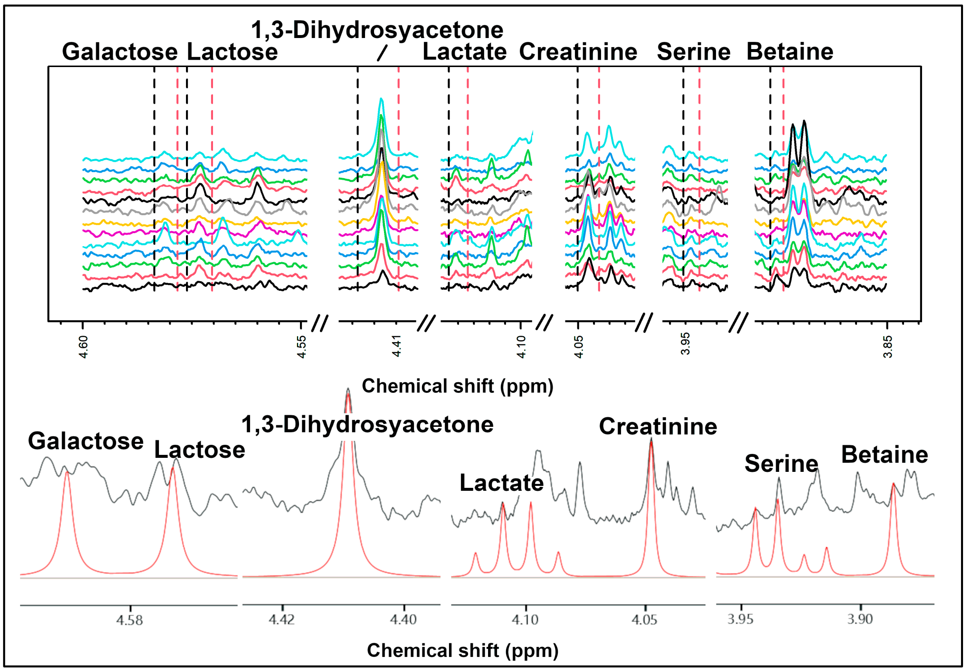

Urine

Figure S12

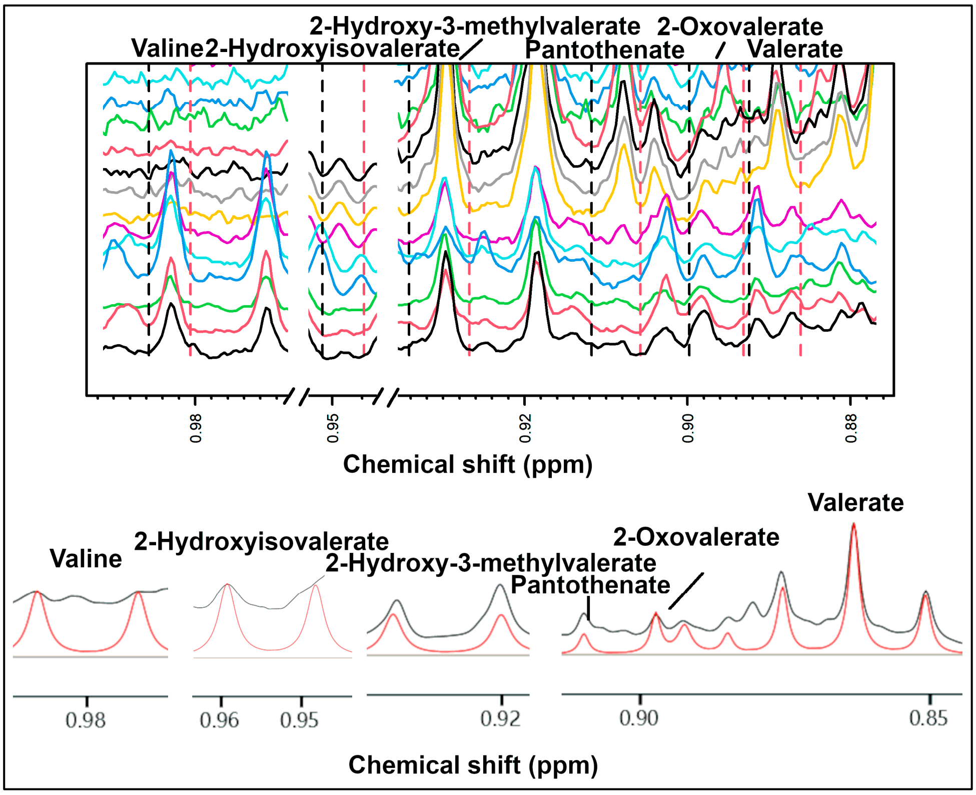

Figure S13

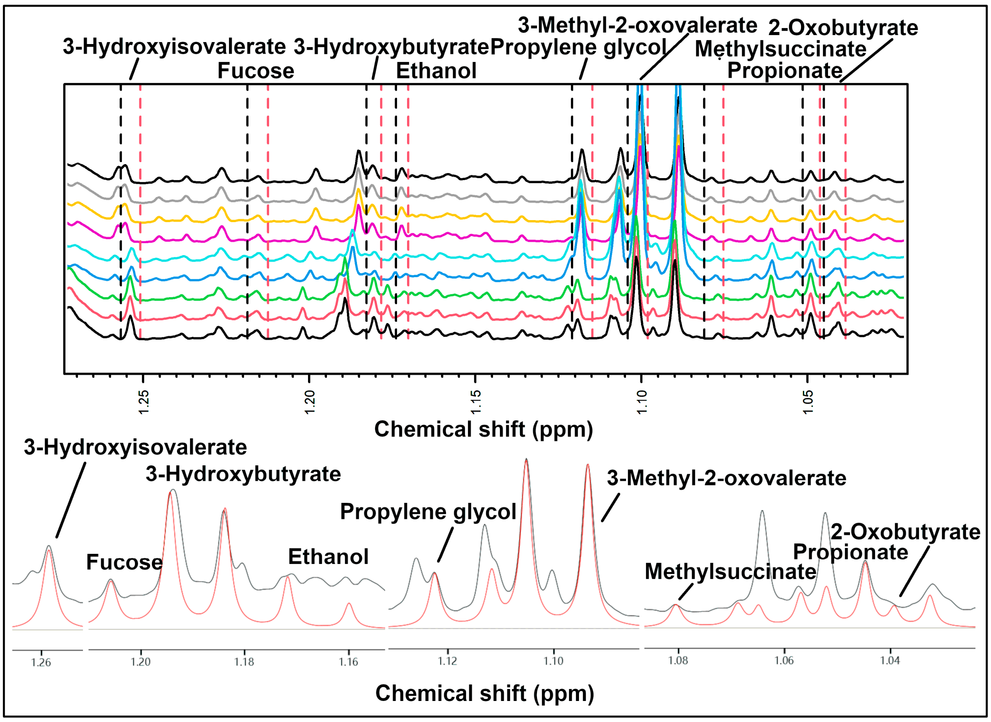

Figure S14

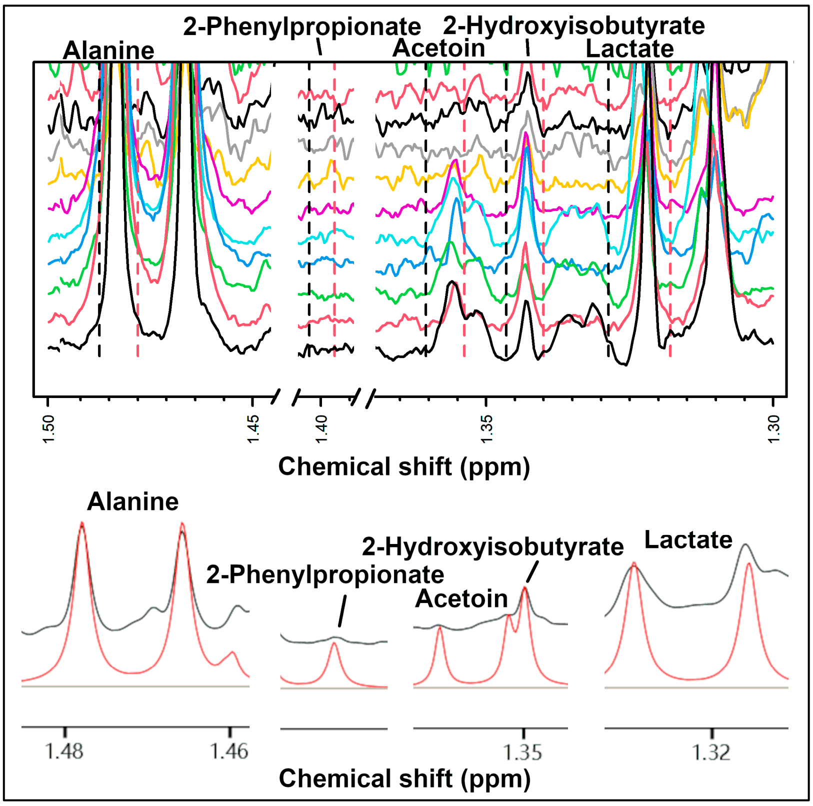

Figure S15

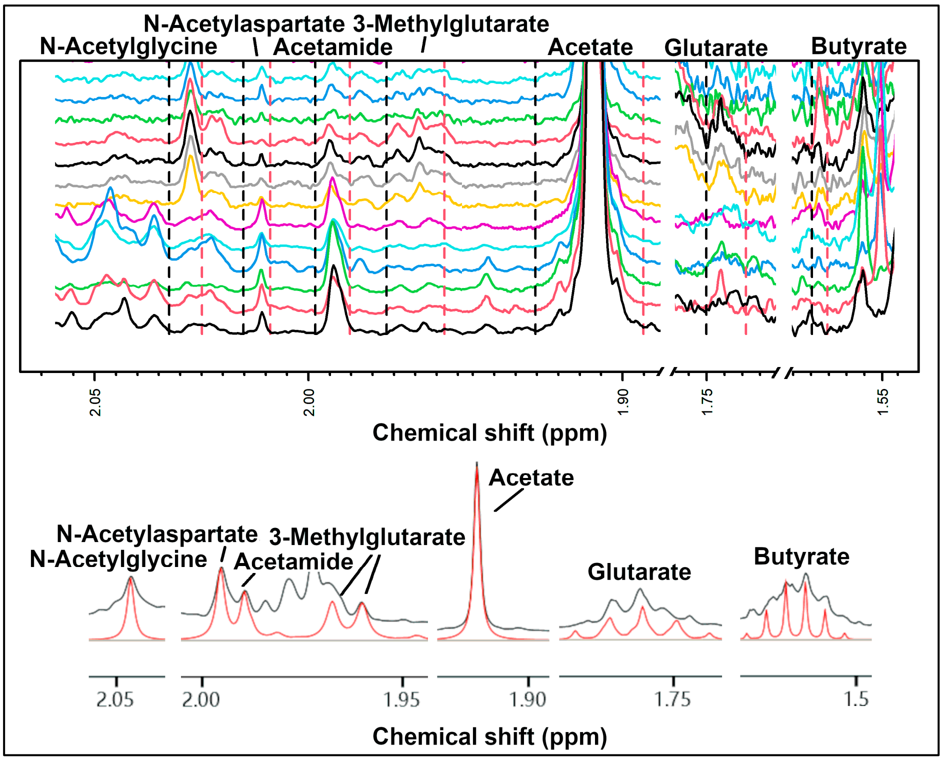

Figure S16

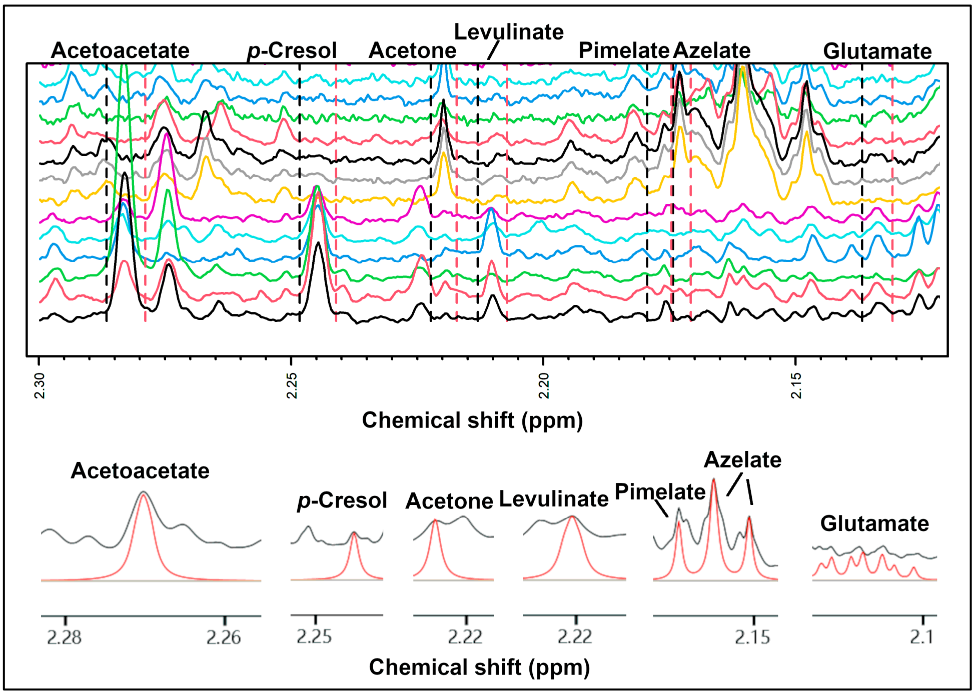

Figure S17

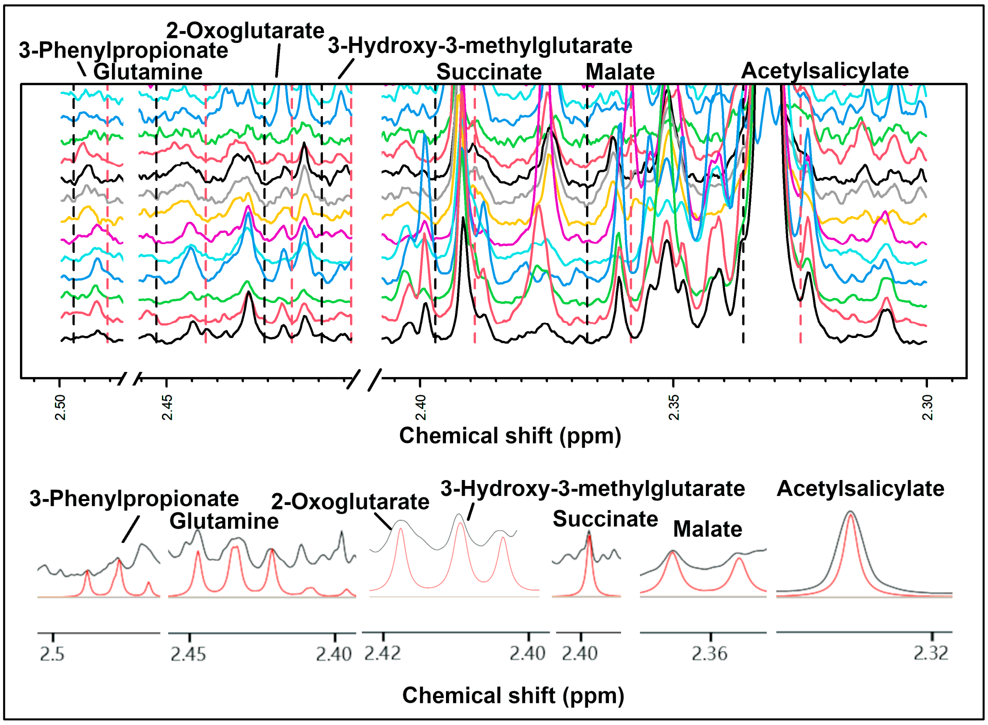

Figure S18

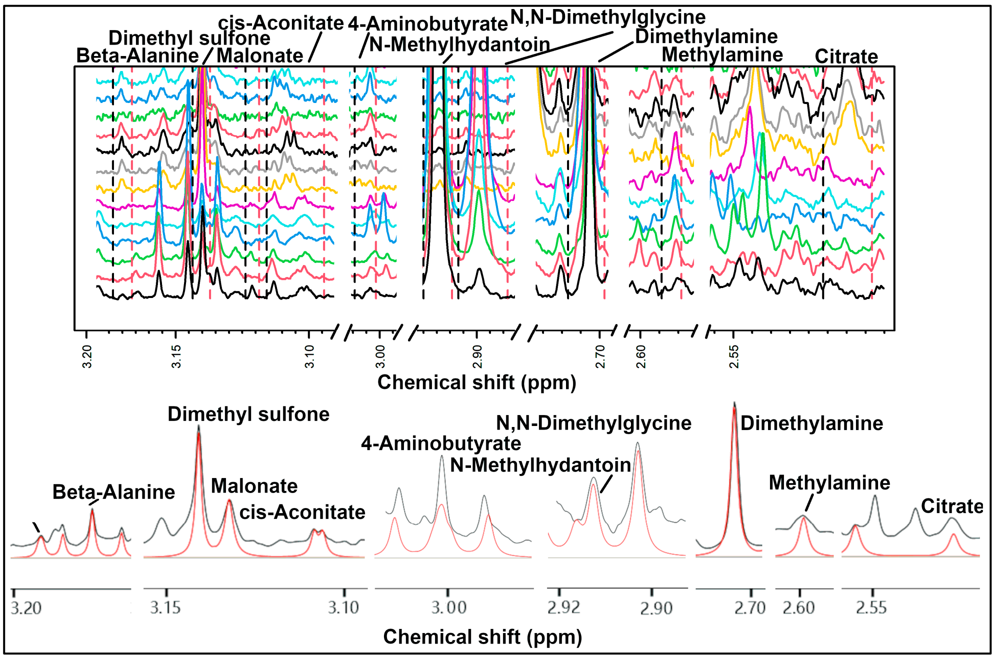

Figure S19

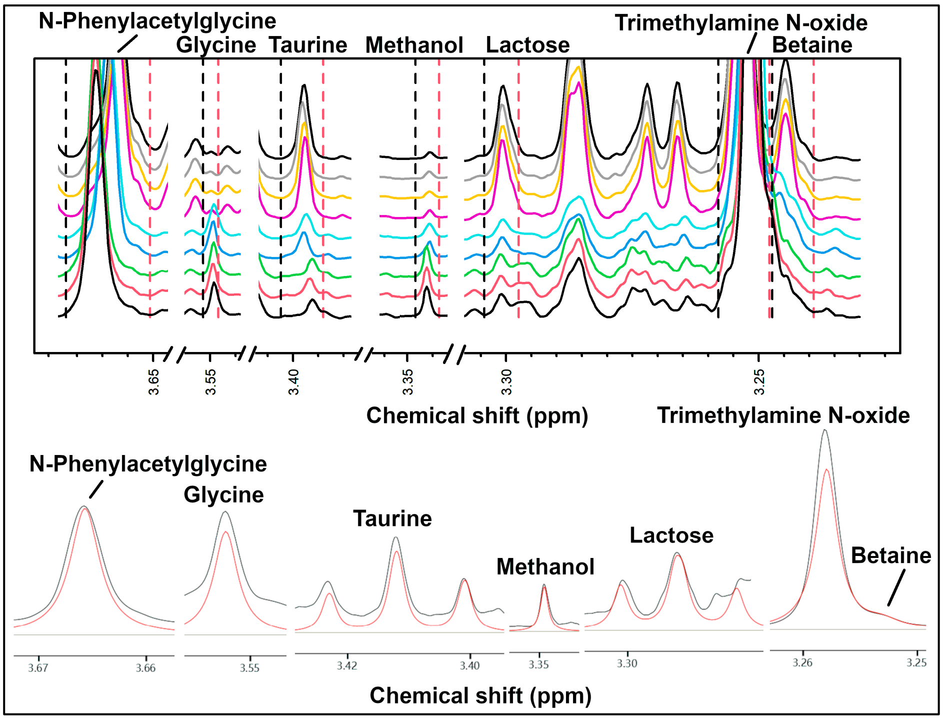

Figure S20

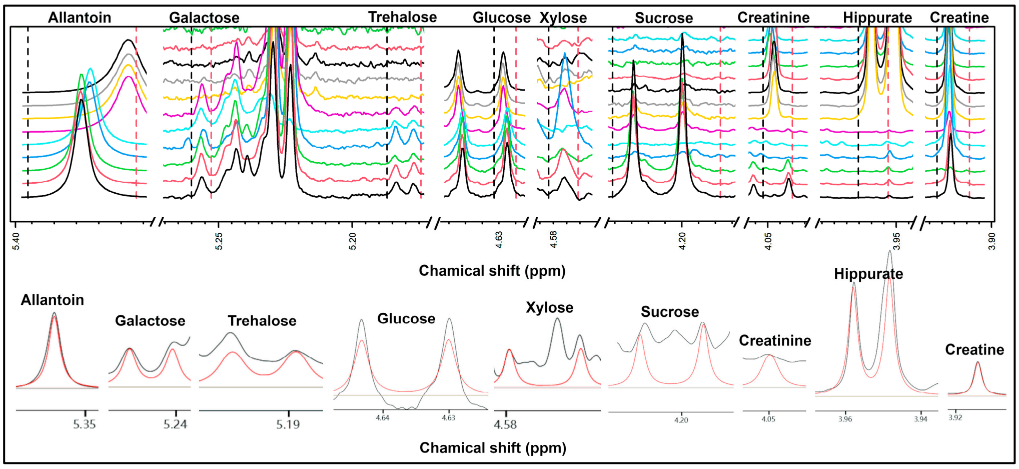

Figure S21

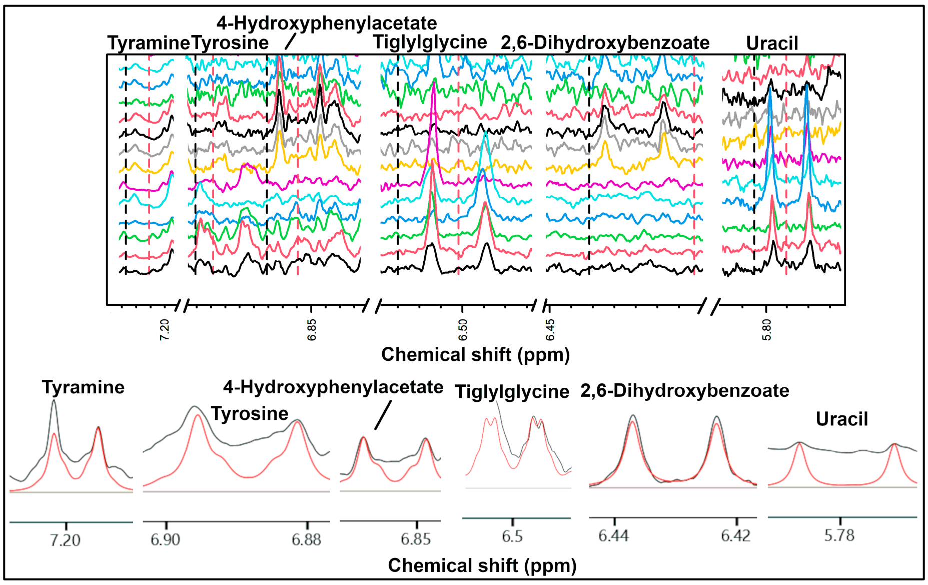

Figure S22

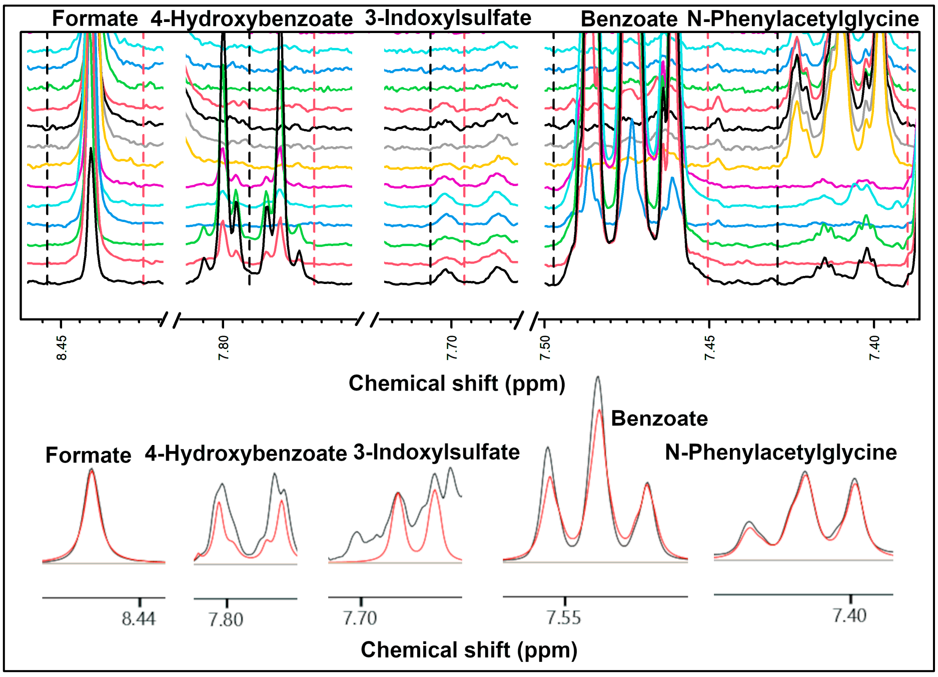

Serum

Figure S23

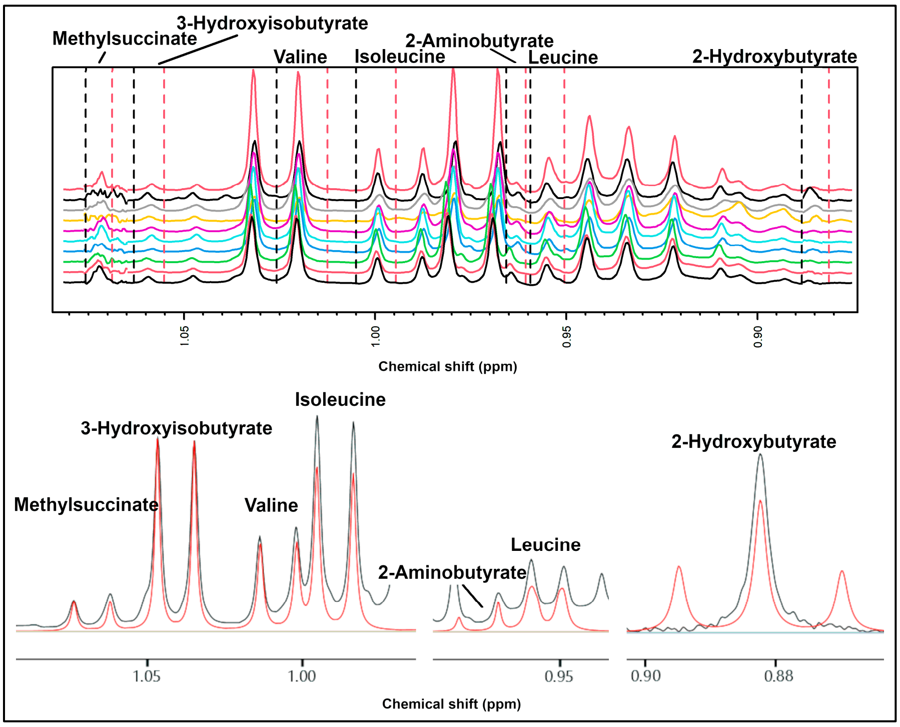

Figure S24

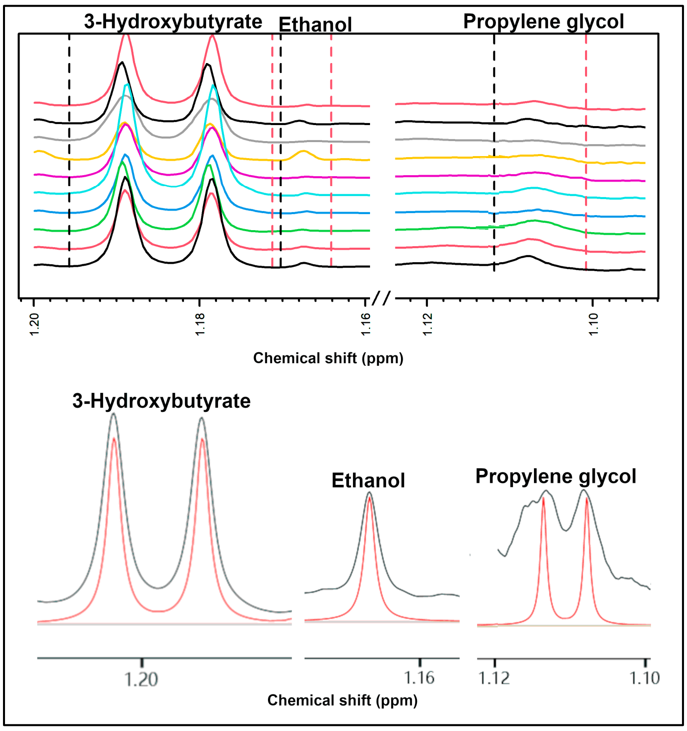

Figure S25

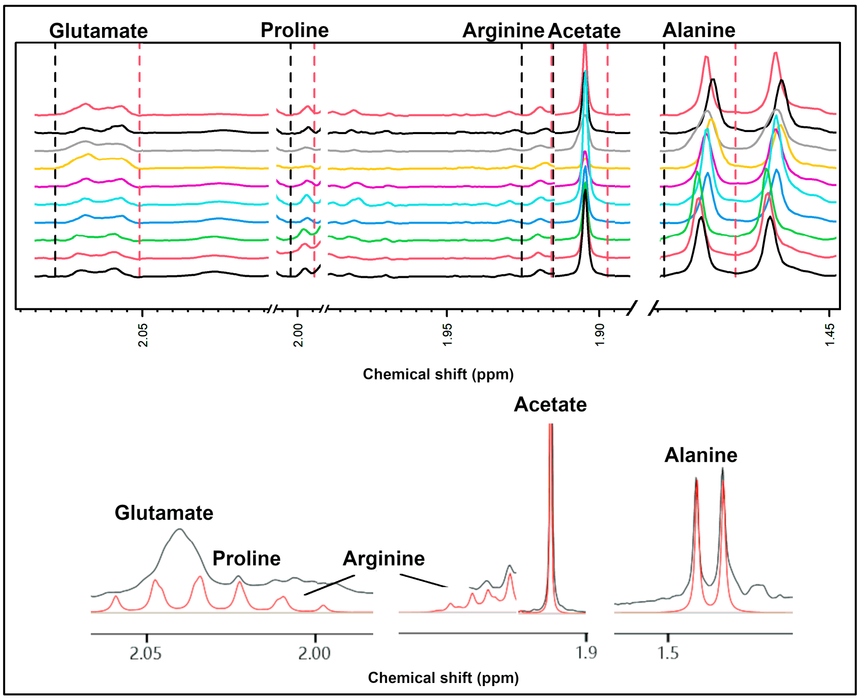

Figure S26

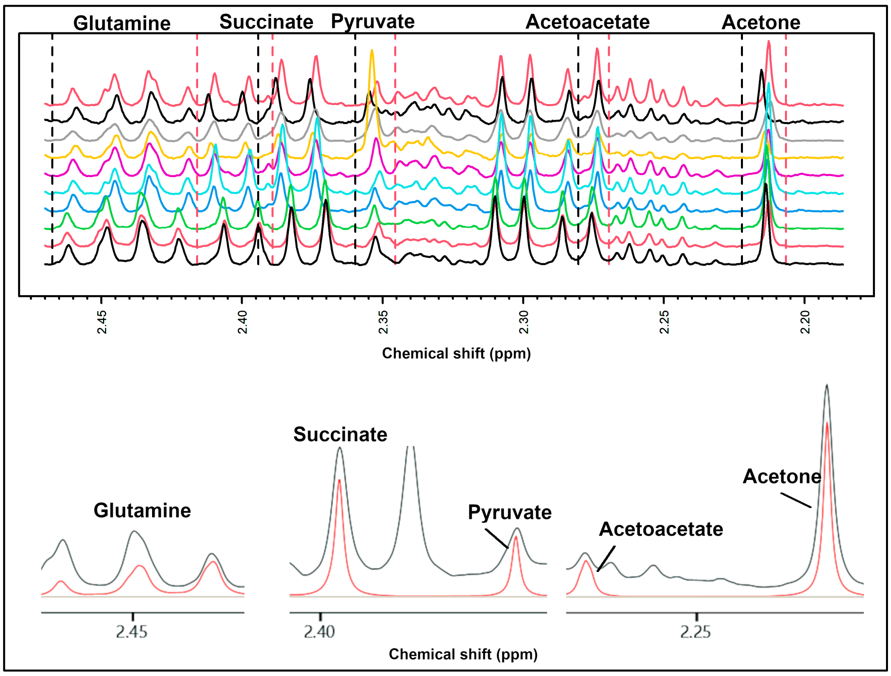

Figure S27

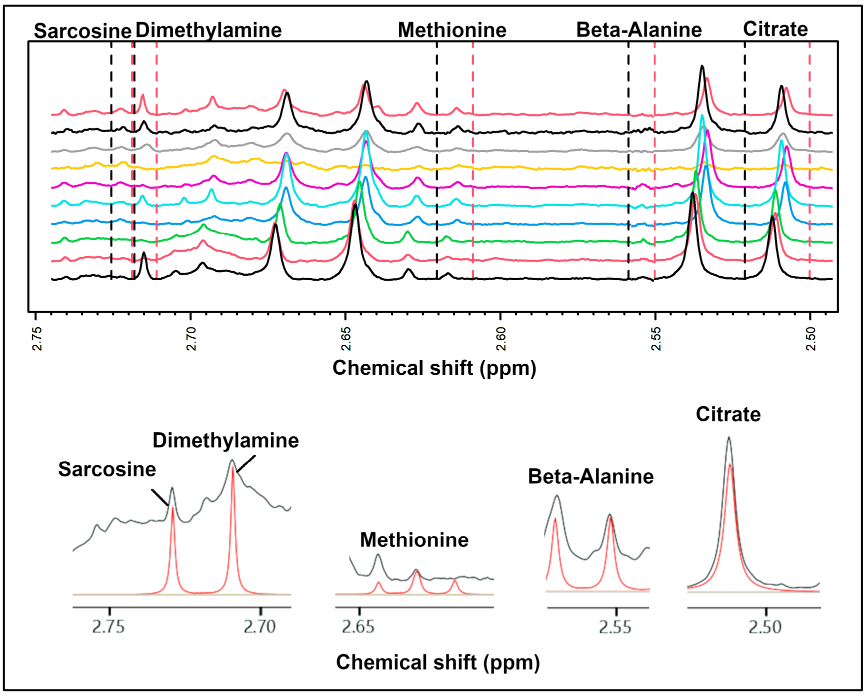

Figure S28

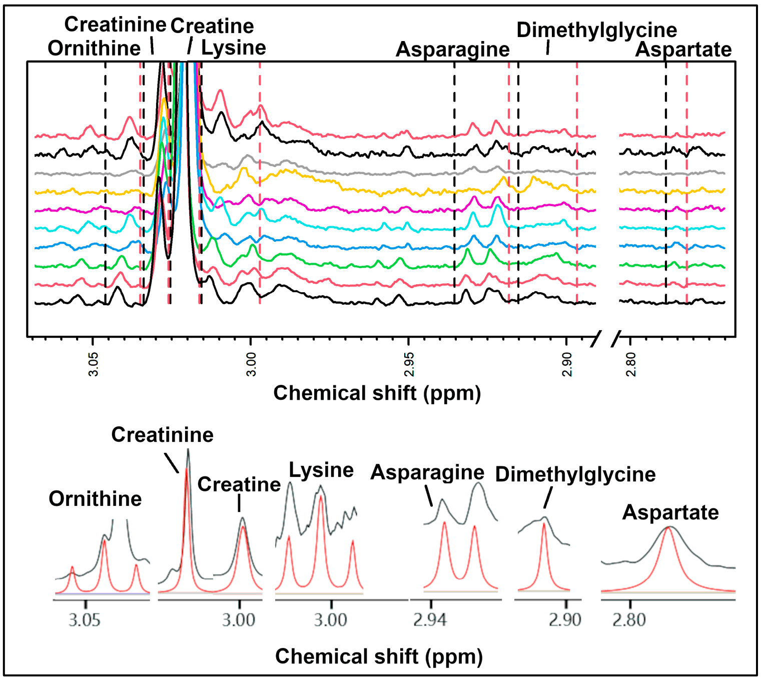

Figure S29

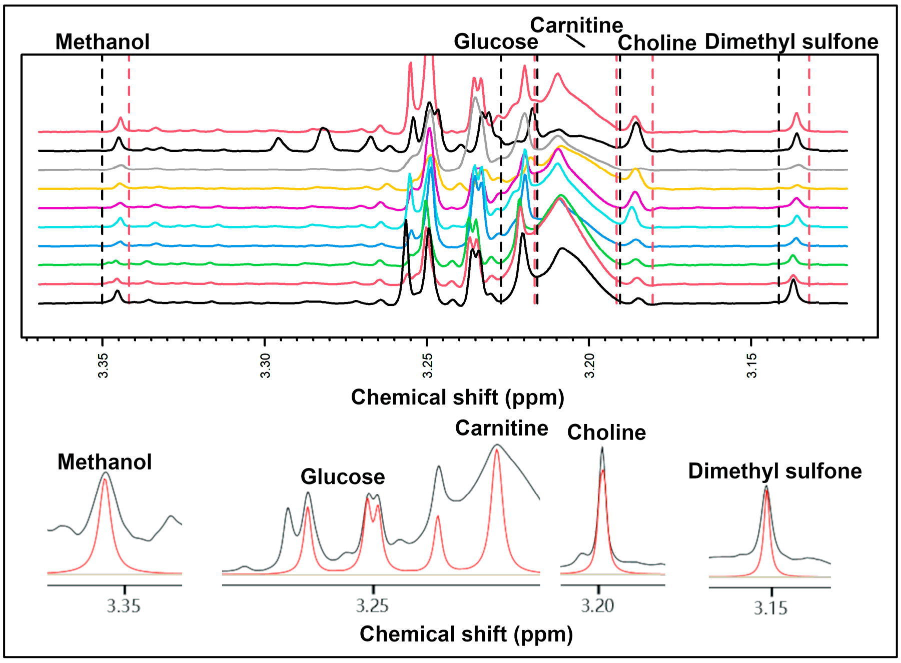

Figure S30

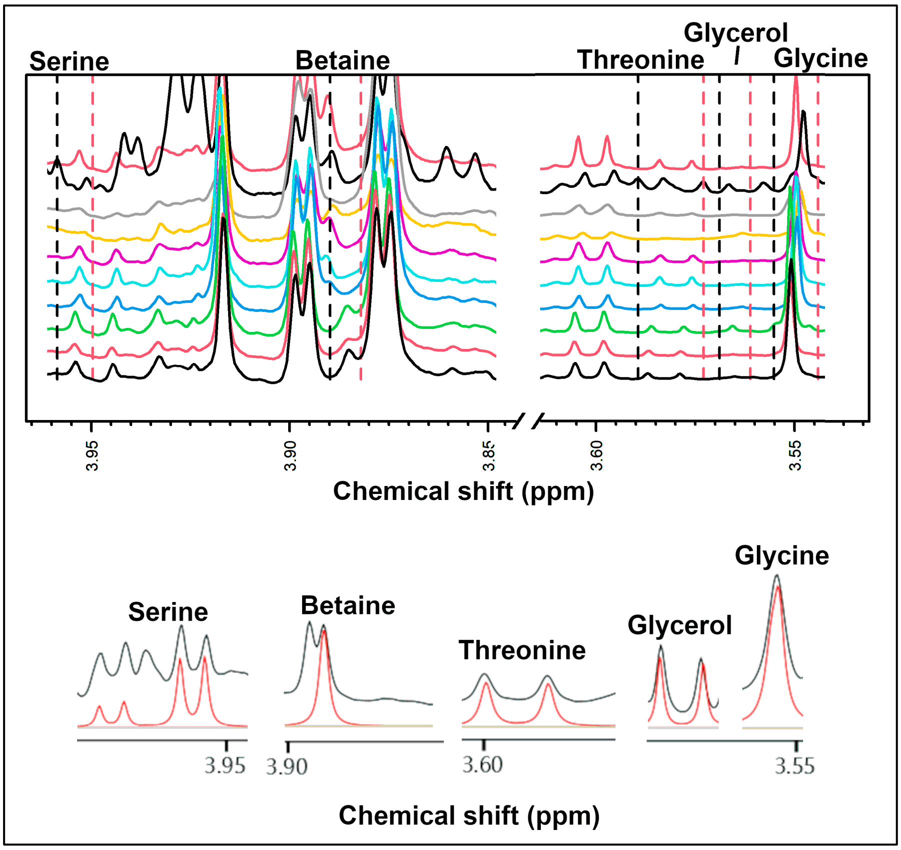

Figure S31

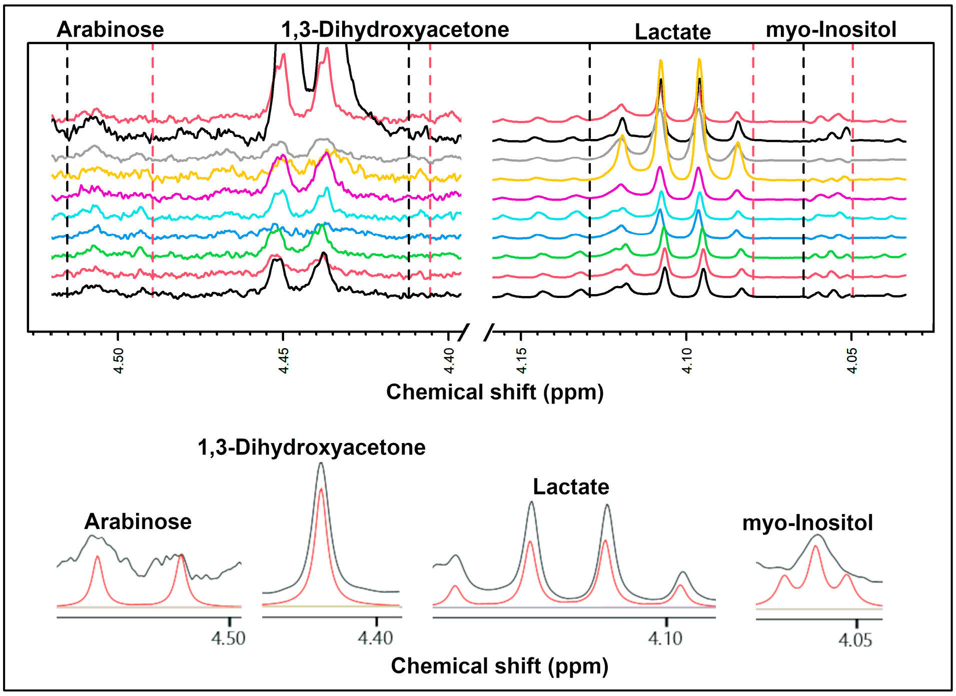

Figure S32

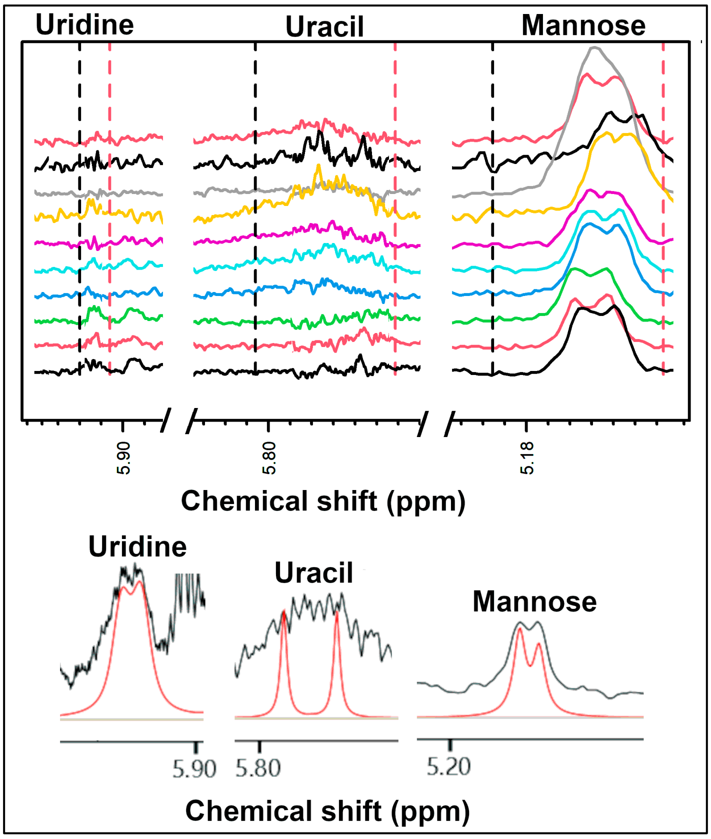

Figure S33

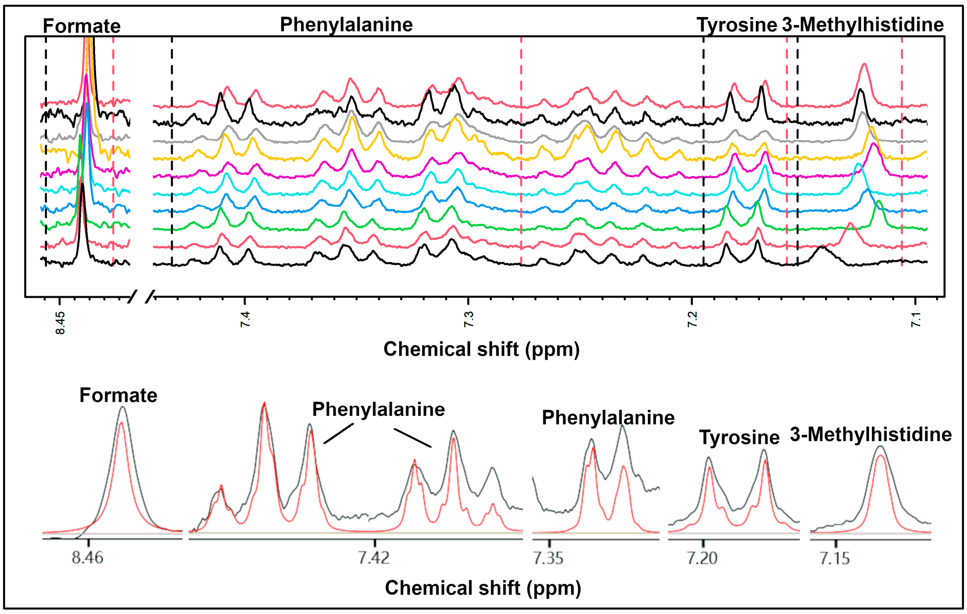
Formate
Phenylalanine
Tyrosine
3-Methylhistidine
8.45
7.4
7.3
7.2
7.1
Chemical shift (ppm)
Formate
Phenylalanine
Phenylalanine
Tyrosine
3-Methylhistidine
8.46
7.42
7.35
7.20
7.15
Chemical shift (ppm)
